# Comparison of the Population Genetic Structures of *Borrelia* Virulence Gene *ospC* and a Putatively Neutral Intergenic Spacer

**DOI:** 10.1002/ece3.74634

**Published:** 2026-11-01

**Authors:** Józefina Wasilewska, Joanna Różańska‐Wróbel, Jacek Radwan

**Affiliations:** ^1^ Evolutionary Biology Group, Faculty of Biology Adam Mickiewicz University Poznań Poland

**Keywords:** balancing selection, *Borrelia afzelii*, *ospC*, population structure

## Abstract

*Borrelia afzelii* , a major causative agent of Lyme borreliosis in Europe, exhibits high polymorphism of the outer surface protein C (*ospC*) gene, which facilitates evasion of the vertebrate immune response. Its extensive polymorphism has been hypothesized to result from balancing selection imposed by host immunity, which predicts reduced population genetic structure at *ospC* relative to neutrally evolving genomic regions. To test this prediction, we genotyped *ospC* and the *rrs–rrl* intergenic spacer (IGS) in *B. afzelii* samples from questing *Ixodes ricinus* ticks and bank voles ( *Clethrionomys glareolus* ) sampled across nine populations in Poland between 2020 and 2024. Among‐population differentiation was consistently lower at *ospC* than at IGS, and while IGS exhibited a significant isolation‐by‐distance pattern, no such signal was detected for *ospC*. Collectively, these findings support the hypothesis that balancing selection maintains *ospC* polymorphism and counteracts geographic differentiation, with implications for the evolutionary epidemiology of Lyme borreliosis in Europe.

## Graphical abstract

*Borrelia afzelii* , a major causative agent of Lyme borreliosis in Europe, exhibits extensive polymorphism at the *ospC* gene, which is hypothesized to be maintained by balancing selection. We investigated whether *ospC* shows reduced population structure compared with the putatively neutral rrs–rrl intergenic spacer (IGS) by analyzing *B. afzelii* isolates from questing *Ixodes ricinus* ticks and bank voles across nine populations in Poland. The lower geographic differentiation and absence of isolation‐by‐distance patterns at *ospC* support the hypothesis that balancing selection contributes to the maintenance of allelic diversity across regions.

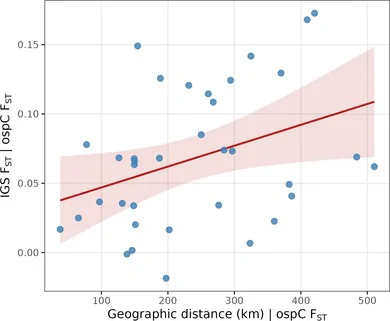

## 1 Introduction

*Borrelia afzelii* is a dominant genospecies within the *Borrelia burgdorferi* sensu lato complex in Europe and constitutes one of the primary causative agents of Lyme borreliosis (Steere et al. 2016). This spirochete bacterium persists through an enzootic transmission cycle involving both a tick vector (Stanek et al. 2012) and a broad range of vertebrate reservoir hosts (Piesman and Gern 2004), primarily small mammals such as rodents and occasionally birds (Mannelli et al. 2012; Heylen et al. 2017). In Europe, *Ixodes ricinus* serves as the principal tick vector responsible for transmission of *Borrelia* spirochetes among vertebrate reservoir hosts (Strnad et al. 2017). The ecological versatility of *B. afzelii* contributes to its extensive geographic distribution across Europe, with particularly high prevalence in Central and Northern regions where *I. ricinus* populations are abundant (Rauter and Hartung 2005).

A key factor facilitating *Borrelia* infection is its outer surface protein C (OspC), encoded by the *ospC* gene located on the cp26 plasmid (Byram et al. 2004; Tilly et al. 2006). OspC is expressed during the initial phase of mammalian infection, during which it plays a critical role in host colonization, but at the same time it is one of the first antigens recognized by the vertebrate immune system (Barbour and Travinsky 2010). The *ospC* gene is remarkably polymorphic, with numerous divergent alleles coexisting within local *Borrelia* populations; alleles belonging to different major groups differ by ~20% at the nucleotide level on average (Wang et al. 1999). This extensive level of genetic diversity has been widely documented in multiple studies across *B. burgdorferi* sensu lato complex (Wang et al. 1999; Brisson and Dykhuizen 2004; Barbour and Travinsky 2010; Girard et al. 2011), including *B. afzelii* (Bunikis et al. 2004; Schüler et al. 2015).

The extensive *ospC* polymorphism observed across the *B. burgdorferi* sensu lato complex is widely attributed to balancing selection, supported by allelic divergence predating the split of *Borrelia* genospecies and by the distribution of *ospC* allele frequencies within local populations being significantly more even than expected under a neutral model (Wang et al. 1999; Qiu et al. 2002). Two mechanisms have been proposed to explain this pattern. The first was proposed by Brisson and Dykhuizen (2004), who demonstrated non‐random associations between *ospC* major groups and different North American reservoir host species. The authors argued that interspecific multiple‐niche polymorphism is alone sufficient to maintain *ospC* diversity. The second invokes negative frequency‐dependent selection, which is proposed to be driven by strain‐specific host immunity, whereby common variants are preferentially cleared from immunized hosts (Wang et al. 1999; Barbour and Travinsky 2010; Haven et al. 2011). In *B. afzelii* specifically, signals of balancing selection have previously been detected at *ospC* in bank vole populations (Różańska‐Wróbel et al. 2024). The same study found that specific MHC Class II alleles predicted IgG antibody levels against particular OspC variants among seropositive individuals, suggesting that host immune‐gene variation may modulate, though likely does not solely drive, the maintenance of OspC polymorphism.

One of the important consequences of balancing selection is that a locus under such selection should exhibit reduced population genetic structure (lower among‐population differentiation) compared to neutrally evolving genomic regions (Schierup et al. 2000; Muirhead 2001; Fijarczyk and Babik 2015). However, local differences in selection pressures may lead to the reverse patterns, with infectivity genes showing higher than expected genetic structure (Anderson et al. 2005; Kawecki and Ebert 2004). In the case of *Borrelia*, such local adaptation might stem, for example, from different composition of locally available hosts. Thus, spatial patterns of distribution of genetic variation might reflect the conflicting pressures from balancing selection versus local adaptation. Investigating these patterns can thus inform us on the relative strength of these two evolutionary forces. In *Borrelia* several studies compared genetic structure of OspC with that of *rrs–rrl* intergenic spacer (IGS), a non‐coding chromosomal region expected to be subject to predominantly neutral evolution (Attie et al. 2007; Margos et al. 2011). Margos et al. (2008) and Qiu et al. (2008) found that *ospC* shows less clear geographic structure than putatively neutral markers, consistent with balancing selection maintaining ancient polymorphism. However, only Qiu et al. (2008) formally quantified this pattern, and did so only between continents, leaving it unclear whether it also holds within continents or at smaller spatial scales. This is an important gap, as understanding at which spatial scale a significant inter‐population genetic differentiation can arise is important not only to infer the mode of selection on OspC, but also for OspC‐based vaccine design (Di et al. 2022) or for understanding of tissue dissemination potential of local *Borrelia* strains, given this potential is linked to OspC variants carried by different strains (Dykhuizen et al. 2008; Wormser et al. 2008). Here, we use AMOVA to determine the proportion of variance in frequencies of *ospC* and IGS distributed within and between populations separated by dozens to hundreds of kilometers. Furthermore, we investigate, for the first time to our knowledge, if population differentiation at both markers increased with geographic distance. We also test the prediction that because of balancing selection acting on OspC, differentiation at this marker will be significantly less predicted by geographic distance compared to putatively neutral IGS.

## 2 Materials and Methods

### 2.1 Study System and Sampling

Bank voles (412) and questing ticks (1003) were sampled between 2020 and 2024 in deciduous forests across 9 populations in Poland (Table 1, Figure 1), under approval of the Local Ethics Committee for Animal Experimentation in Poznań (decision no. 35/2021). The sampling schedule was determined by logistical constraints, but care was taken to avoid confounding spatial and temporal variation. Thus, in most sampling years (except for 2022 when only one population was sampled), we covered a wide geographic range (Table 1). We also included a sample of ticks from Białowieża, which we received courtesy of Dr. Nicolas De Pelsmaeker. Ear samples and ticks were collected and stored in ethanol at −20°C. Ear biopsies were selected as ticks preferentially attach there, making it a hotspot for detecting tick‐borne pathogens (Cayol et al. 2018; Zawada et al. 2020). The stages of ticks collected in this study included nymphs and adults, as these stages play a critical role in pathogen transmission. Questing larvae were excluded because they generally remain uninfected prior to their first blood meal (Kurtenbach et al. 2006; Pospisilova et al. 2019).

**TABLE 1:** Number of bank vole and tick samples collected and processed for DNA extraction, by population and sampling year (2020–2024).

Population	Years sampled	Bank voles (*n*)	Ticks (*n*)	Total
Augustów	2020, 2022	34	43	77
Białowieża	2024	0	314	314
Brok	2020, 2022, 2023	88	35	123
Długosiodło	2023	32	47	79
Goły Jon	2020, 2023, 2024	35	141	176
Grobka	2022, 2023	126	140	266
Niezgoda	2020, 2023	24	59	83
Puszcza Zielonka	2020, 2022	31	140	171
Urwitałt	2021	42	84	126
Total		412	1003	1415

**FIGURE 1:**
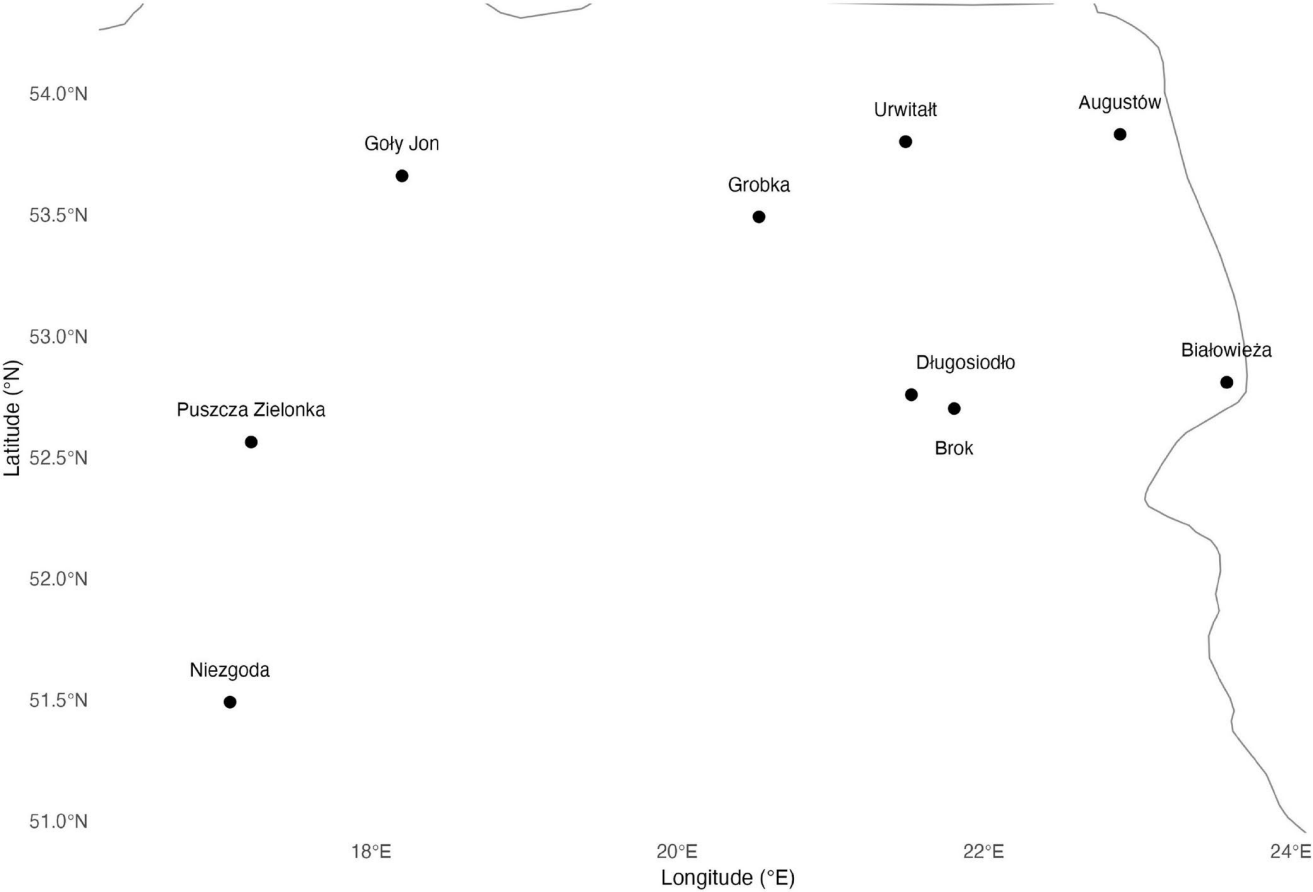
Geographic distribution of nine studied populations across Poland. Each point represents a population sampling site, with coordinates given in decimal degrees (latitude, longitude): Augustów (53.79620° N, 23.15076° E), Białowieża (52.74799° N, 23.77519° E), Brok (52.70028° N, 21.91884° E), Długosiodło (52.76377° N, 21.63235° E), Goły Jon (53.69369° N, 18.15199° E), Grobka (53.51667° N, 20.62469° E), Niezgoda (51.50806° N, 17.05649° E), Puszcza Zielonka (52.58318° N, 17.15235° E), Urwitałt (53.80851° N, 21.65682° E). These coordinates indicate a single reference point for each population area; in reality, samples were collected within the surrounding region rather than at the exact coordinate. Sampling within a population extended over an area of up to 1 km. Map lines delineate study areas and do not necessarily depict accepted national boundaries.

### 2.2 Detection and Genotyping of *Borrelia afzelii*

DNA was extracted from 1003 tick samples using the Syngen DNA Mini Kit (Syngen Biotech), following the manufacturer's protocol for tick tissue, and from 412 vole samples using the NucleoMag Tissue Kit (Macherey‐Nagel), following the manufacturer's protocol for DNA purification from cells and tissue. *Borrelia* infection status of all samples was determined by nested‐PCR that targets the IGS region of *Borrelia afzelii* , located between the *rrs* (encoding 16S rRNA) and *rrl* (encoding 23S rRNA) genes. The nested‐PCR was performed as described by Bunikis et al. (2004), with the first PCR using primers F (5′‐GTATGTTTAGTGAGGGGGGTG‐3′) and R (5′‐GGATCATAGCTCAGGTGGTTAG‐3′) at an annealing temperature of 56°C for 35 cycles. The second PCR used primers Fn (5′‐AGGGGGGTGAAGTCGTAACAAG‐3′) and Rn (5′‐GTCTGATAAACCTGAGGTCGGA‐3′) at an annealing temperature of 60°C for 35 cycles. PCRs were run with Type‐it Multiplex PCR Master Mix (Qiagen, Hilden, Germany) using 1 μL of template DNA and 1 μL of each primer (total volume 25 μL). Each plate contained a negative control. Individuals showing a visible PCR band on agarose gels were considered *Borrelia*‐positive, and these samples were subsequently sequenced by the Sanger method to confirm and identify the *Borrelia* species present. The obtained Sanger sequences were compared to reference sequences in the NCBI database using BLAST. Samples identified as *Borrelia afzelii* based on sequence similarity were selected for further analysis.

Based on the obtained sequences, a new forward primer was designed (5′‐ATCATmTCCTTTCTAAGAGGAAGATAA‐3′) for use with a reverse internal primer described by Bunikis et al. (2004). Using these two primers, barcode‐tagged primers were designed incorporating barcodes described by Kozich et al. (2013) to enable identification of unique samples. The barcode‐tagged primers were then employed to amplify the IGS fragment from all infected ticks and bank voles in a PCR reaction comprising 35 cycles with an annealing temperature of 59°C. The reaction mix contained 1 μL of template DNA and 1 μL of each primer in a total volume of 25 μL. Each plate contained a negative control.

For samples identified as positive for *Borrelia afzelii* , barcode‐tagged primers targeting the *ospC* gene were employed. These primers were based on sequences described by Różańska‐Wróbel et al. (2024) and incorporated barcodes as described by Kozich et al. (2013). The barcode‐tagged primers were used to amplify the *ospC* fragment from infected ticks and bank voles in a PCR reaction consisting of 45 cycles with an annealing temperature of 50°C. A lower cycle number (35, as used for IGS) was tested first but yielded substantially fewer *ospC*‐positive amplifications. The cycle number was therefore raised to 45 to ensure successful amplification of low‐abundance *ospC* variants, including in samples with low bacterial loads or mixed infections. This brought the numbers of *ospC*‐ and IGS‐positive samples used in the analyses to comparable levels (177 and 190, respectively).

The reaction mix contained 1 μL of template DNA and 1 μL of each primer, in a total volume of 25 μL. Each plate contained a negative control.

Amplicons were sequenced on an Illumina MiSeq platform using the MiSeq Reagent Kit v2 Nano (paired‐end, 2 × 250 bp). Genotyping, including de‐multiplexing, clustering, and filtering of unique variants, was performed with AmpliSAS software using default Illumina settings (Sebastian et al. 2016). Clustering used a substitution error rate of 1% and an indel error rate of 0.001%, with a minimum‐frequency‐respect‐to‐dominant threshold of 25%. Filtering required a minimum amplicon depth of 100 reads and a minimum per‐amplicon frequency of 3%.

### 2.3 Statistical Analyses

All statistical analyses were performed in R, v. 4.2.3 (R Core Team 2023) using RStudio, 2026.01.1+403 (Posit team 2026), and all data visualization was performed using the ggplot2 package (Wickham 2016). All analyses described below were performed independently for two molecular markers: the *rrs–rrl* IGS and the outer surface protein C gene (*ospC*).

To examine the genetic structure across populations, haplotype networks were constructed using the haplotype() and haploNet() functions from the pegas package (Paradis 2010). Node size in the resulting networks is proportional to haplotype frequency, and the number of lines crossing each branch represents the number of mutational steps separating adjacent haplotypes. Nucleotide diversity (*π*) was calculated with the nuc.div() function (pairwise.deletion = TRUE) and haplotype diversity (Hd) with the hap.div() function, both from the pegas package (Paradis 2010). Tajima's *D* was calculated per population for both loci using the tajima.test function from the same package; *p*‐values were obtained via the beta‐distribution approximation of the *D* statistic (Tajima 1989).

To evaluate whether the two ecological compartments of the transmission cycle differed genetically, an AMOVA with host type (questing ticks vs. bank voles) as the grouping factor was performed. As we found no significant variance between host types (see Section 3), to quantify the partitioning of genetic variation within and among populations, another AMOVA was conducted, with bv and qt pooled. Both AMOVAs used the poppr.amova() function from the poppr package (v. 2.9.6; Kamvar et al. 2014), with the statistical significance of variance components assessed using a permutation test with the randtest() function from the ade4 package (v. 1.7‐23; Dray and Dufour 2007).

Pairwise genetic differentiation between populations was subsequently quantified using *F*_ST_ for haploid data (Weir and Cockerham 1984) via the pairwise.WCfst() function (diploid = FALSE) from the hierfstat package (v. 0.5‐11; Goudet and Jombart 2022). Confidence intervals were estimated from 1000 bootstrap replicates resampling individuals within populations.

Isolation by distance (IBD) was tested using the Mantel test implemented in the mantel() function from the vegan package (v. 2.6‐4; Oksanen et al. 2022), comparing pairwise *F*_ST_ matrix with geographic distance matrix. Geographic distance between populations was calculated as the haversine distance in km (R package geosphere) between population coordinates (one coordinate pair per population; see the legend of Figure 1). To assess whether differentiation patterns at IGS are significantly different from (independent of) *ospC*, a partial Mantel test was performed using the mantel.partial() function from the vegan package (v. 2.6‐4; Oksanen et al. 2022).

## 3 Results

A total of 12 distinct IGS variants and 13 distinct OspC variants were identified, representing 11 OspC major groups as defined by Wang et al. (1999), with variant nomenclature following Strandh and Råberg (2015) (Table 2).

**TABLE 2:** OspC variants detected in this study with GenBank accession numbers of corresponding sequences follow those adapted by Strandh and Råberg (2015).

OspC variant	OspC major group	GenBank accession number	Number of cases
7b	7	X81523.1	77
9c	9	JX103493.1	64
10b	10	JN828675.1	46
3a	3	AY363712.1	41
4c	4	JN828672.1	24
19a	19	—	16
2a	2	AY363711.1	16
1b	1	FJ750333.1	13
12	12	AY150205.1	12
Z135	S	U91793.1	6
7d	7	—	2
1a	1	AY363710.1	2
14a	14	FJ546548	1

The haplotype networks based on IGS and *ospC* sequences showed that individual variants at both loci were broadly shared among populations (Figure 2). Several rare variants at both loci were restricted to one or two populations. *ospC* haplotypes were separated by larger mutational distances between adjacent variants. Nucleotide diversity was markedly higher for *ospC* than for IGS, whereas haplotype diversity was similar between the two markers. Tajima's *D* was positive in all nine populations for both loci; it was higher for *ospC* than for IGS in eight of nine populations, and was significantly positive in three of nine populations for *ospC* and in one of nine for IGS (Appendix 1). Among samples classified as single infections at both loci, comparison of *ospC* and IGS types revealed substantial concordance, although the association was not strictly one‐to‐one. While several combinations were consistently observed, some *ospC* types were associated with more than one IGS type. Conversely, certain IGS types were found in association with more than one *ospC* type.

**FIGURE 2:**
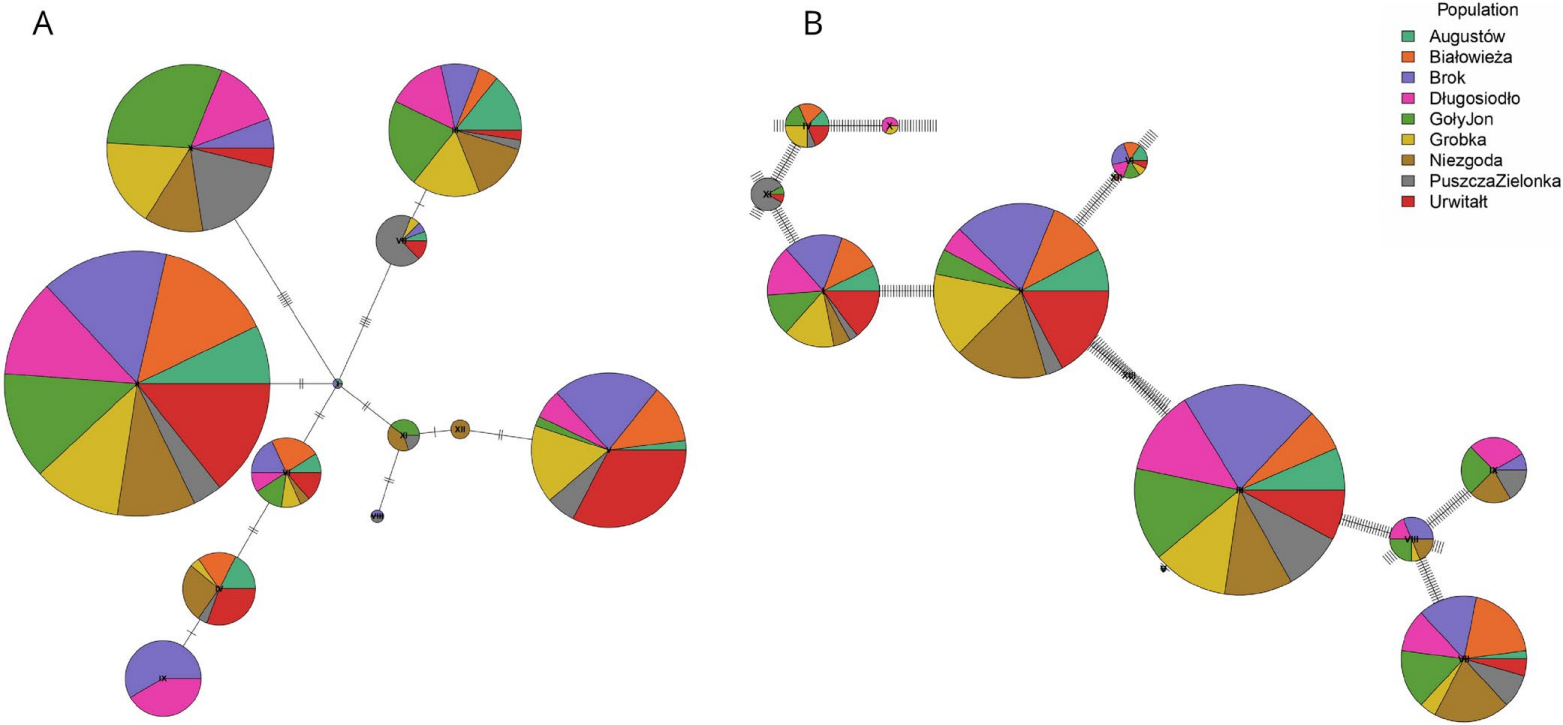
Haplotype networks of *B. afzelii* IGS and *ospC* gene variants identified in nine populations across Poland. In both networks, node size is proportional to haplotype frequency, and the number of lines crossing each branch represents the number of mutational steps between adjacent haplotypes. Pie charts within nodes represent the proportion of individuals from each population, color‐coded as indicated in the legend. (A) Twelve IGS haplotypes identified among 336 sequences (effective sequence length 363 bp; nucleotide diversity *π* = 0.0147; haplotype diversity Hd = 0.860). (B) Thirteen *ospC* haplotypes identified among 320 sequences (effective sequence length 333 bp; nucleotide diversity *π* = 0.173; haplotype diversity Hd = 0.854).

Host type (questing ticks vs. bank voles) accounted for less than 0.3% of total genetic variation at either locus (*p* = 0.24), with 97.77% of variation distributed within rather than between host groups. Pairwise *F*_ST_ ranged from −0.0002 to 0.189 for IGS (mean 0.068) and from −0.033 to 0.109 for *ospC* (mean 0.034) (Table 3). AMOVA revealed that the vast majority of genetic variation was distributed within rather than among populations at both loci. For the IGS marker, 93.30% of total variation resided within populations, with only 6.70% attributable to differences among populations (*p* = 0.001). Even more pronounced pattern was observed for *ospC*, where within‐population variation accounted for 96.76% of the total, with only 3.24% partitioned among populations (*p* = 0.001).

**TABLE 3:** Pairwise *F*_ST_ values among nine *Borrelia afzelii* populations calculated using (a) the IGS marker and (b) the *ospC* marker, with 95% confidence intervals shown below each estimate.

(a)								
	Augustów	Białowieża	Brok	Długosiodło	Goły Jon	Grobka	Niezgoda	Puszcza Zielonka
Białowieża	0.0565 [−0.0118, 0.2330]	—	—	—	—	—	—	—
Brok	0.0495 [0.0115, 0.1671]	0.0118 [−0.0005, 0.1009]	—	—	—	—	—	—
Długosiodło	0.0360 [0.0042, 0.1442]	0.0648 [0.0220, 0.1799]	0.0089 [−0.0081, 0.0941]	—	—	—	—	—
Goły Jon	0.1438 [0.0725, 0.2924]	0.1624 [0.0961, 0.2917]	0.1151 [0.0544, 0.2266]	0.0533 [0.0059, 0.1746]	—	—	—	—
Grobka	0.0922 [0.0297, 0.2203]	0.0784 [0.0232, 0.1974]	0.0571 [0.0153, 0.1574]	0.0416 [0.0012, 0.1503]	0.0098 [−0.0137, 0.1192]	—	—	—
Niezgoda	0.0688 [0.0196, 0.2180]	0.0509 [0.0210, 0.1576]	0.0277 [0.0097, 0.1110]	0.0143 [−0.0054, 0.1223]	0.0240 [−0.0081, 0.1367]	0.0094 [−0.0052, 0.0945]	—	—
Puszcza Zielonka	0.1535 [0.0915, 0.2880]	0.1886 [0.1194, 0.3146]	0.1334 [0.0696, 0.2492]	0.0823 [0.0279, 0.2035]	−0.0002 [−0.0107, 0.0958]	0.0077 [−0.0094, 0.1215]	0.0500 [0.0100, 0.1705]	—
Urwitałt	0.1231 [0.0266, 0.2862]	0.0114 [−0.0185, 0.1545]	0.0324 [−0.0033, 0.1505]	0.0958 [0.0347, 0.2254]	0.1452 [0.0797, 0.2714]	0.0449 [0.0035, 0.1595]	0.0584 [0.0222, 0.1744]	0.1464 [0.0789, 0.2791]

(b)								
	Augustów	Białowieża	Brok	Długosiodło	Goły Jon	Grobka	Niezgoda	Puszcza Zielonka
Białowieża	0.0111 [−0.0210, 0.1568]	—	—	—	—	—	—	—
Brok	−0.0093 [−0.0197, 0.0982]	0.0144 [−0.0089, 0.1129]	—	—	—	—	—	—
Długosiodło	0.0391 [0.0114, 0.1627]	0.0372 [0.0132, 0.1278]	0.0155 [0.0004, 0.0972]	—	—	—	—	—
Goły Jon	0.0386 [0.0051, 0.1686]	0.0208 [0.0025, 0.1174]	0.0125 [−0.0023, 0.0929]	−0.0125 [−0.0124, 0.0525]	—	—	—	—
Grobka	−0.0325 [−0.0300, 0.0914]	0.0185 [−0.0112, 0.1498]	0.0091 [−0.0061, 0.0935]	0.0457 [0.0127, 0.1501]	0.0532 [0.0149, 0.1607]	—	—	—
Niezgoda	0.0337 [0.0035, 0.1556]	0.0080 [−0.0015, 0.0948]	0.0035 [−0.0106, 0.0841]	0.0147 [−0.0026, 0.0960]	0.0102 [−0.0051, 0.0982]	0.0403 [0.0101, 0.1334]	—	—
Puszcza Zielonka	0.0900 [0.0372, 0.2381]	0.0712 [0.0301, 0.1863]	0.0922 [0.0378, 0.2095]	0.0535 [0.0189, 0.1611]	0.0361 [0.0046, 0.1458]	0.0955 [0.0460, 0.2122]	0.0926 [0.0407, 0.2178]	—
Urwitałt	−0.0267 [−0.0349, 0.1224]	0.0223 [−0.0164, 0.1594]	0.0266 [−0.0016, 0.1279]	0.0757 [0.0338, 0.1932]	0.0795 [0.0293, 0.2032]	−0.0209 [−0.0224, 0.0731]	0.0555 [0.0199, 0.1629]	0.1085 [0.0506, 0.2457]

*Note:* Values represent the lower triangle of the pairwise distance matrix. Dashes (—) indicate redundant upper triangle cells.

The Mantel test revealed that the two markers differ in the degree of IBD (Figure 3). For *ospC*, only a weak and non‐significant positive correlation between pairwise *F*_ST_ and geographic distance was detected (*r* = 0.270, *p* = 0.109). For IGS, a moderate and statistically significant positive correlation was found (*r* = 0.414, *p* = 0.024). Partial Mantel tests revealed that the difference in IBD patterns was statistically significant, with IGS showing isolation when genetic differentiation at *ospC* was accounted for (partial *r* = 0.363, *p* = 0.039) (Figure 4).

**FIGURE 3:**
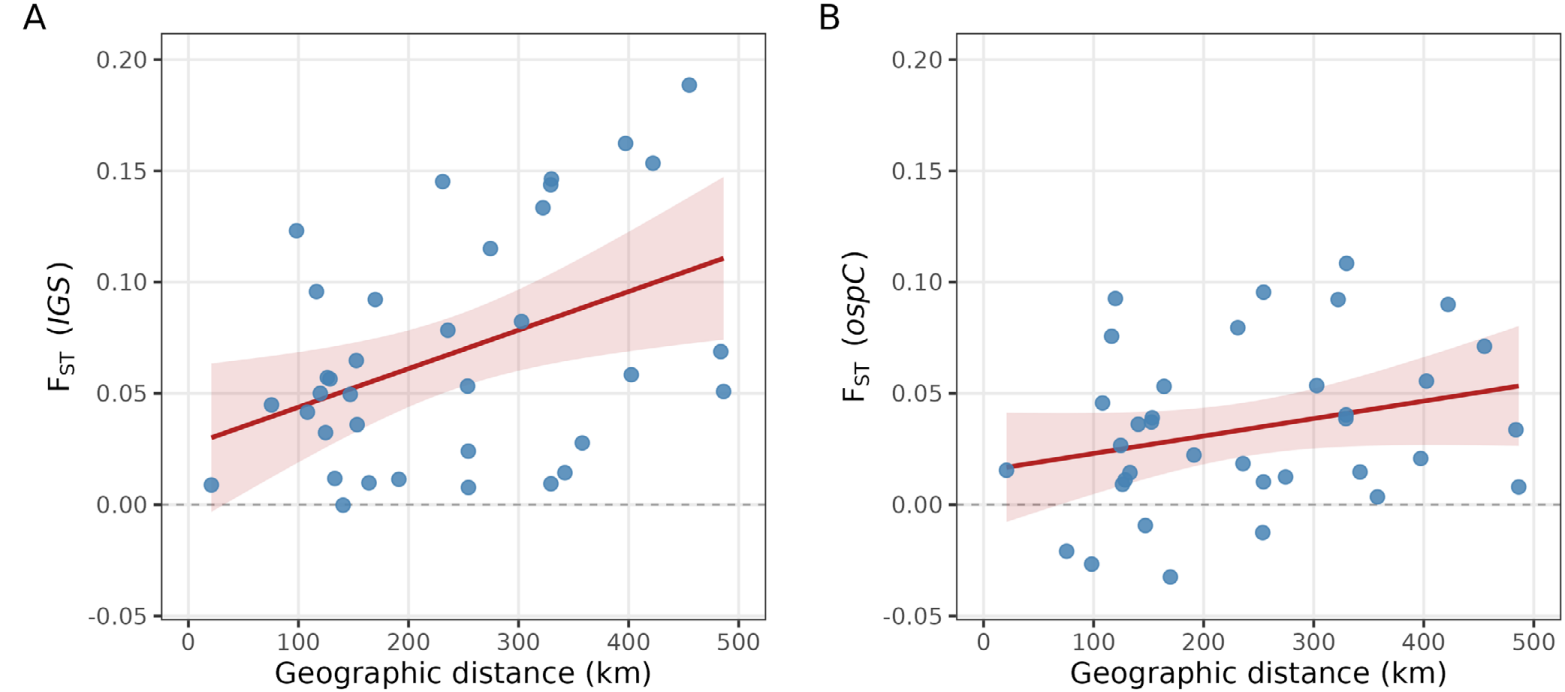
Mantel tests between pairwise *F*_ST_ and geographic distance. (A) IGS *F*_ST_ versus geographic distance (Mantel *r* = 0.414, *p* = 0.024). (B) *ospC F*_ST_ versus geographic distance (Mantel *r* = 0.270, *p* = 0.109). Each point represents one pairwise population comparison. The red line shows a linear regression fit with a 95% confidence interval. Statistical significance was assessed using 9999 permutations.

**FIGURE 4:**
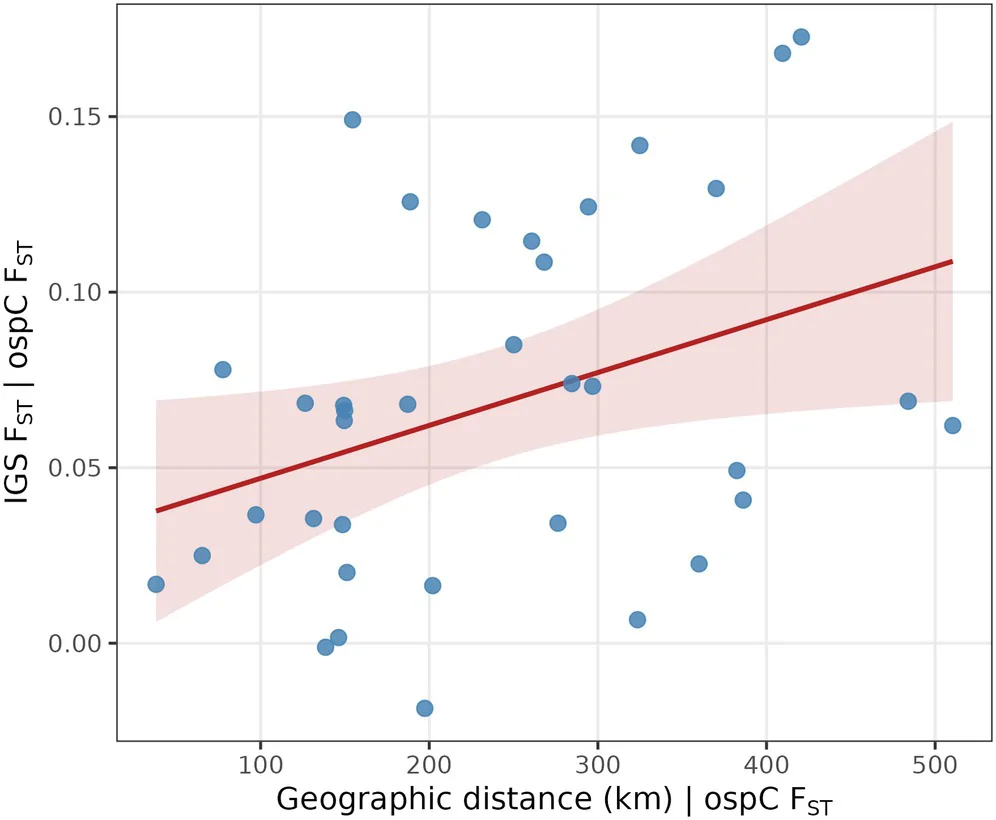
Partial Mantel test between pairwise *F*_ST_ and geographic distance. Correlation between IGS *F*_ST_ and geographic distance after controlling for *ospC F*_ST_ (partial *r* = 0.363, *p* = 0.039). Each point represents one pairwise population comparison. Axes represent residuals from the regression of each distance matrix on the control matrix. The red line shows a linear regression fit with 95% confidence interval. Statistical significance was assessed using 9999 permutations.

## 4 Discussion

This study compared genetic structuring of OspC with that of a presumably neutral *rrs–rrl* IGS marker. While *Borrelia* reproduction is clonal, plasmids are often transmitted horizontally (Qiu et al. 2004; Brisson et al. 2012; Seifert et al. 2015), creating a degree of independence of plasmid‐based markers, as we also documented here. The comparison showed that the *ospC* locus exhibited lower genetic differentiation among populations and a significantly weaker IBD signal compared to the IGS, supporting the predominant role of balancing selection acting on *ospC* polymorphism and counteracting the geographic structuring expected under genetic drift and limited dispersal. Below we discuss these findings in detail.

Both loci displayed substantial allelic diversity, with 12 IGS variants and 13 OspC variants belonging to 11 major OspC groups identified across 9 Polish populations. While more PCR cycles used for OspC amplification (45 vs. 35 for IGS) might potentially have led to inflation of polymorphism via sequencing artifacts, we countered this inflation by applying strict artifact filtering criteria (see Section 2). Indeed, polymorphism level of *ospC* diversity we detected is in line with previous studies of *B. afzelii* in Europe (Bunikis et al. 2004; Pérez et al. 2011; Różańska‐Wróbel et al. 2024) and with reports of extensive *ospC* polymorphism across the broader *B. burgdorferi* sensu lato complex (Wang et al. 1999; Brisson and Dykhuizen 2004; Barbour and Travinsky 2010). Although the two markers showed considerable concordance, with particular IGS variants tending to co‐occur with specific OspC types, this association was not strictly one‐to‐one, consistent with pervasive horizontal gene exchange (Haven et al. 2011). A comparison of allele linkage observed in the present study with that reported by Bunikis et al. (2004) and Hellgren et al. (2011) (Table 4) confirms a degree of non‐independence between the two loci. Our findings are consistent with Hellgren et al.'s (2011) interpretation that the apparent linkage structure in *B. afzelii* may largely reflect the epidemic spread of certain haplotypes rather than a complete absence of recombination. The occurrence of recombination is particularly relevant in the context of balancing selection, as it allows *ospC* alleles to be maintained independently of the genomic background, facilitating their persistence across diverse genetic lineages. This mechanism is supported by the findings of Haven et al. (2011), who demonstrated through whole‐genome analysis of *B. burgdorferi* s.s. followed by simulations that pervasive, localized recombination acting in tandem with negative frequency‐dependent selection at the *ospC* locus can maintain sympatric genomic diversity of *Borrelia* strains. Their simulations indicate that while recombination prevents complete clonal divergence, strong selection on *ospC* drives the clustering of genomic lineages associated with major *ospC* variants.

**TABLE 4:** Comparison of linkage between alleles at *ospC* and IGS observed in Bunikis et al. (2004), Hellgren et al. (2011), and the present study (individuals that were found to have single infections at both loci).

Bunikis et al. (2004)		Hellgren et al. (2011)		This study	
*ospC*	*IGS*	*ospC*	*IGS*	*ospC*	*IGS*
1	1	1	1, 2		
		1b	1	1b	1
2	2	2	2, 1		
3	3	3	3	3a	3
4	4				
		4b	4	4c	4
5	5				
6	6				
7a/7b	7	7a	8, 4, 11	7b	7, 4
8	8				
9	9	9	9, 1	9c	9, 3, 8
		10	3, 3b, 12	10b	3
		13	13		
		15	8		
				19a	2
				B.burg‐Z136	2

The degree of linkage of the two markers resulting from clonal reproduction may explain why haplotype networks for both markers were similarly characterized by broad geographic sharing of common variants, with very few rare population‐specific, private haplotypes. Such pattern might result from two factors. Firstly, high gene flow among sampled localities, reflecting the strong dispersal potential of *I. ricinus* and/or its vertebrate hosts, may lead to such homogenization. Secondly, the pattern may reflect balancing selection on OspC maintaining polymorphism within populations and shaping linked genome‐wide patterns of diversity (Haven et al. 2011). In this context, it is important to note that the *ospC* network displayed higher divergence of *ospC* alleles relative to IGS. This pattern, also noted by Margos et al. 2008, is consistent with long‐term maintenance of divergent alleles by balancing selection (Haven et al. 2011). Positive Tajima's *D* values at both loci are likely to reflect their linkage due to predominantly clonal reproduction, but higher *D* values for *ospC* than for IGS in eight of nine populations suggest that balancing selection acts on *ospC*, with IGS frequencies equalized due to linkage with *ospC*. The Tajima's *D* estimates for *ospC* in our study were similar to those reported by Różańska‐Wróbel et al. (2024) for samples from bank vole populations from North‐Eastern Poland (range *D* = 1.62–2.69).

Further support for balancing selection on OspC comes from the comparison of genetic structure and IBD of the two markers, which, owing to a degree of their independence, we found to vary. First, the proportion of among‐population variation estimated by AMOVA was approximately twofold lower for *ospC* than for IGS, indicating that *ospC* alleles are distributed more homogeneously across populations, which is consistent with balancing selection acting on OspC. Second, IBD was significant for IGS but not for OspC. Furthermore, IGS IBD remained significant even after accounting for the *ospC* distance matrix in the partial Mantel test. While we designed sampling to avoid confounding spatial and temporal variation (see Section 2), Białowieża, located at the eastern edge of our sampling range, was an (opportunistic) exception, as this was the only population sampled in 2024. However, *F*_ST_ between Białowieża and other populations was within range of other population pairs (Table 3), indicating that temporal variation played little role, in line with previous findings (Różańska‐Wróbel et al. 2024). In any case, any temporal bias would affect both markers simultaneously, and thus we conclude that, for IGS, gene flow decreased with increasing geographic separation, but for OspC, even geographically distant populations had similar allele frequencies, a hallmark of balancing selection (Garrigan and Hedrick 2003). A comparable pattern has been documented at major histocompatibility complex (MHC) loci and other immune‐related genes, where balancing selection homogenizes allelic variation across populations, effectively overriding the structuring effects of drift (Strand et al. 2012; Herdegen‐Radwan et al. 2021). These results are consistent with Margos et al. (2008), who, across a sample spanning North America and Europe, observed that the *ospC* gene tree shows deeper branching and less clear geographic structure than IGS and MLST trees, at both inter‐continental and intra‐continental scales. They attributed this pattern to balancing selection maintaining ancient polymorphism, though without quantifying among‐population differentiation. Such quantification was later performed by Qiu et al. (2008), who applied AMOVA to IGS and *ospC* across North American and European samples and found that IGS showed clear geographic differentiation between continents consistent with genetic drift, whereas *ospC* showed low geographic differentiation despite substantial sequence variability, mirroring our own AMOVA and IBD results. However, their comparison was limited to two populations from different continents, leaving open the question of whether such patterns also hold at finer spatial scales where gene flow is plausible. By quantifying this pattern among nine populations separated by tens to hundreds of kilometers, the present study extends Qiu et al.'s (2008) finding to a spatial scale at which it had not previously been tested and shows that the pattern is not limited to inter‐continental comparisons.

No significant partitioning of genetic variation was detected between host types (< 0.3% of total variation, *p* = 0.24), suggesting that questing ticks and bank voles sampled within the same locality harbored similar assemblages of IGS and *ospC* variants. Under the multiple‐niche polymorphism model, in which different host species represent distinct selective environments favoring different *ospC* major groups (Brisson and Dykhuizen 2004), bank voles, as a single host species, would be expected to exhibit a distinct subset of *ospC* variants relative to questing ticks. Our results do not support this prediction, but are in agreement with the results of Råberg et al. (2017), who showed that OspC frequencies do not differ among the three main rodent hosts: bank voles, yellow‐necked mice, and shrews. This suggests that, unlike in *Borrelia burgdorferi* sensu stricto, OspC polymorphism is not associated with *Borrelia* host specificity. However, we acknowledge that the absence of such differentiation may reflect the dominant role of *Clethrionomys glareolus* as the principal reservoir of *B. afzelii* in the sampled population, although we find this explanation unlikely given that yellow‐necked mice and shrews were common in our sampling sites (unquantified personal observations).

In conclusion, this study provides empirical support for the balancing selection hypothesis in *B. afzelii* , demonstrating that *ospC* exhibits lower population genetic structure and a significantly weaker IBD signal of IBD than the IGS marker. These results contribute to a growing body of evidence that balancing selection plays a central role in maintaining the extensive polymorphism observed at *ospC* across the *B. burgdorferi* sensu lato complex. Future studies should test specific mechanisms causing this balancing selection. Our results furthermore highlight the importance of contrasting selectively constrained and putatively neutral markers when interpreting population genetic patterns in vector‐borne pathogens.

## Author Contributions

**Józefina Wasilewska:** conceptualization (supporting), data curation (lead), formal analysis (lead), investigation (equal), methodology (equal), visualization (lead), writing – original draft (lead), writing – review and editing (equal). **Joanna Różańska‐Wróbel:** formal analysis (supporting), investigation (equal), methodology (equal), writing – review and editing (equal). **Jacek Radwan:** conceptualization (lead), formal analysis (supporting), funding acquisition (lead), investigation (equal), methodology (equal), project administration (lead), supervision (lead), writing – review and editing (equal).

## Funding

This work was supported by Narodowe Centrum Nauki (UMO‐2019/34/A/NZ8/00231).

## Conflicts of Interest

The authors declare no conflicts of interest.

## Data Availability

The data that support the findings of this study are openly available in the OSF repository at https://doi.org/10.17605/OSF.IO/WSEUH. Raw sequencing reads have also been deposited in the European Nucleotide Archive (ENA) under project accession PRJEB127466. A key for assigning sequences to individual samples, based on embedded tag barcodes, is available at the associated OSF project, folder “Demultiplexing barcode keys.”

## Appendix 1 Tajima's *D* Statistics Calculated for (a) IGS and (b) *ospC*

**Table:**

Population	Tajima's *D*	*p* (beta)
(a)		
Augustów	0.8177	0.43453
Białowieża	1.2168	0.24539
Brok	1.6987	0.10504
Długosiodło	1.7374	0.09608
Goły Jon	2.0066	0.05379
Grobka	2.2341	0.02953
Niezgoda	1.9023	0.06731
Puszcza Zielonka	1.8045	0.08303
Urwitałt	1.3583	0.19401
(b)		
Augustów	1.1362	0.28197
Białowieża	2.3517	0.02009
Brok	2.0581	0.04861
Długosiodło	2.1333	0.03904
Goły Jon	2.0231	0.05149
Grobka	1.4299	0.17192
Niezgoda	1.9865	0.05584
Puszcza Zielonka	1.8281	0.07817
Urwitałt	1.5614	0.13537
